# Potential Role of Dietary Antioxidants During Skin Aging

**DOI:** 10.1002/fsn3.70231

**Published:** 2025-05-01

**Authors:** Nicla Tranchida, Francesco Molinari, Gianluca Antonio Franco, Marika Cordaro, Rosanna Di Paola

**Affiliations:** ^1^ Department of Chemical, Biological, Pharmaceutical and Environmental Sciences University of Messina Messina Italy; ^2^ Department of Veterinary Sciences University of Messina Messina Italy; ^3^ Department of Biomedical, Dental and Morphological and Functional Imaging University of Messina Messina Italy

**Keywords:** antioxidants, diet, nutrition, oxidative stress, skin aging

## Abstract

The skin is the largest organ of the human body in contact with the outside world. Different are its functions: body temperature regulation, mechanical barrier, coating, and sensory activity are just to name a few. Like any other part of the human organism, it too undergoes the phenomenon of aging, a complex biological process classified as chronological aging. Although this is a natural and inevitable physiological process, certain external factors have been shown to have an important impact. One of these, much discussed in recent years, is diet. The relevance of diet to the clinical features of skin aging, particularly the biochemical and histological changes that occur in it, is now well established. Lately, there has been a growing focus on which foods can be considered skin‐friendly and which cannot. Açai berries, *Moringa oleifera* , and spirulina are just some of the emerging nutrients that counteract skin aging because of their potent antioxidant properties. Conversely, foods high in trans‐fatty acids, refined sugars are related to accelerated skin aging as they are associated with the production of advanced glycation end products. Due to the interest generated in recent years on the subject, a subspecialty of anti‐aging medicine called “nutricosmetics” has even emerged. This review aims to highlight the studies emerging in the last five years regarding what can be considered “skin‐friendly” foods in contrast to what can be considered “skin‐unfriendly” habits, taking into consideration studies regarding the innovations recently developed in terms of nutrients and skin aging strategies.

## Graphical abstract

Nutrition plays an important role in maintaining youthful and healthy skin. Incorrect eating habits can cause an acceleration of skin aging. Functional bioactive compounds in foods can help slow down the skin aging process.

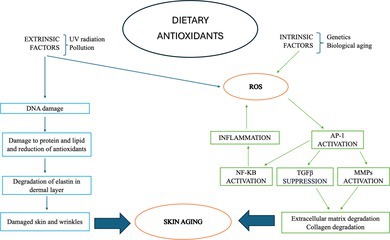

## 1 Introduction

The skin, which accounts for approximately 15% of the adult body's total weight, is incredibly versatile (Sarkar and Das 2021).

Skin is waterproof, adaptable, and protective (Mohamed and Hargest 2022). The three layers of an animal's skin—the dermis, epidermis, and subcutaneous tissue—protect it from various hazards, including chemical exposure, harmful materials, UV radiation, mechanical and thermal injuries, and infections (Woo 2019).

Medical cosmetology, active chemicals, and physical photoprotection methods are used for treating and preventing various skin disorders like photoaging (Geng et al. 2021). In recent years, the significance of proper nutrition for maintaining healthy skin has gained increased recognition, with certain nutrients emerging as potential alternatives to traditional methods of treating photoaging symptoms.

Unhealthy diets negatively affect all skin biological processes, resulting in various skin diseases from youth to old age (Ahmed and Mikail 2024). Nutrition and food influence health, but health issues can lead to nutrient deficiencies. Maintaining a balanced diet contributes to skin healing and preservation. Unhealthy diets have a detrimental effect on all skin biological processes from youth to aging, and can cause some skin diseases (Ahmed and Mikail 2024).

The body can adapt to environmental changes through physiological and psychological means. Over the course of aging, there is an increased risk of mortality, deterioration of physiological functions, decreased tissue regeneration, and mounting macromolecular damage (Lee et al. 2021). The primary characteristics of this condition include increasing mortality risk, progressive loss of physiological function integrity, diminished tissue regeneration, and accumulation of macromolecular damage (Lee et al. 2021). The interplay between internal and external factors can cause aging (Woo 2019).

Photoaging is a type of aging that specifically targets body parts exposed to light (Geng et al. 2021; Chen, Zhao, et al. 2022). While skin ages chronologically around the body, photoaging, as the name implies, affects body parts exposed to light (Geng et al. 2021; Chen, Zhao, et al. 2022).

The skin reveals a person's age and overall health status. The best way to reduce the impact of extrinsic skin aging processes is “prevention” (Hart et al. 2019). Combining sunscreen use, a nutritious diet rich in antioxidants, and a self‐care routine consisting of exercise and calorie restriction is vital for preventing skin aging (Choi 2020).

Diet significantly influences the production or activation of reactive oxygen species (ROS), which is the main factor associated with extrinsic skin aging (Chen et al. 2021).

Ultraviolet radiation triggers cell signaling through ROS production. This pathway then prompts the expression of anti‐oxidative enzymes like catalase and superoxide dismutase, which have anti‐aging and cell‐protective properties, as well as shielding cells from dangerous ROS levels (Lim et al. 2022).

The body generates advanced glycation end products (AGEs) due to high sugar intake (Mohiuddin 2019; Zheng et al. 2022). Advanced glycation end products (AGEs) hinder collagen fiber reconstruction, thereby accelerating the aging process.

Omega‐3 fatty acids with mono‐ and polyunsaturated structures are inversely correlated with severe photoaging (Solà Marsiñach and Cuenca 2019). Antioxidants such as vitamins A, C, and E, carotenoids, flavonoids, resveratrol, curcumin, and green tea polyphenols can effectively decelerate this process (Mumtaz et al. 2021).

Coenzymes of metallothioneins and glutathione, zinc, selenium, and copper, lower intracellular oxidative stress and regulate skin protection (Pincemail and Meziane 2022). A wise diet and well‐balanced nutrition are necessary to delay aging and extend life.

In light of this, the aim of this review is to highlight new studies that have emerged in recent years regarding new trends in the food sector and how these impact skin aging. The most recent findings regarding how diet can accelerate and/or slow down the skin aging process will be examined (Zohoori 2020).

The method used was characterized by accurate bibliographic research concerning publications on the correlation between nutrition and skin aging, in particular, in the last five years.

For this purpose, several databases were consulted, such as PubMed, Google Scholar, Web of Science, and Scopus, using relevant keywords including “skin aging”, “food”, and “nutrition”.

The findings presented in this review may represent a valuable resource for people who wish to mitigate the effects of age on their skin through conscious dietary choices.

## 2 Skin Structure and Function

**FIGURE 1:**
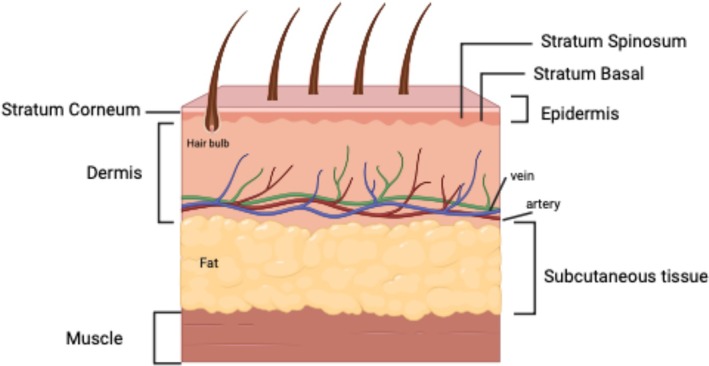
Structure of the skin.

The epidermis is the uppermost layer, the dermis is the middle layer, and the subcutaneous tissue is the lowest layer (Figure 1).

The epidermis, the outermost layer of the skin, regulates skin color and functions as a protective barrier against moisture loss.

The dermis, located beneath the epidermis, contains hair follicles, blood vessels, lymphatic vessels, sweat glands, and connective tissue (Lee et al. 2021).

The deeper subcutaneous layer, referred to as the hypodermis, contains connective tissue and fat.

In thicker skin regions such as the palms and soles, the five epidermal layers—stratum basale, stratum spinosum, stratum granulosum, stratum lucidum, and stratum corneum—are subdivided.

In some areas, the stratum lucidum is absent when the epidermis consists of only four layers (Mohamed and Hargest 2022; Lopez‐Ojeda 2022).

Keratinocytes constitute the majority of the strong and relatively thin epidermis, which is the skin's outermost layer (Feng et al. 2022; Moroki 2023).

The basal layer cells in the epidermis generate new skin cells. Keratinocytes ascend gradually to the surface of the epidermis. Surface cells are continuously supplanted by apparently younger cells advancing from the deeper layers (Koeppen and Niekrash 2021).

The stratum corneum, which is both healthy and waterproof, prevents most foreign bacteria, viruses, and substances from entering the body (Woo 2019; Fodor and Dumitrascu 2020). The outer skin layers, including the epidermis, shield underlying structures such as blood vessels, muscles, internal organs, and nerves.

The palms of the hands and soles of the feet have a significantly thicker stratum corneum than other body parts for enhanced protection (Choi 2019).

One of the main factors influencing skin tone is pigment melanin, which is produced by melanocytes and is dispersed throughout the basal layer of the epidermis (Venkatesh et al. 2019). Melanin shields DNA against harmful UVR, thereby averting the risks of skin cancer and skin aging (Ferrara et al. 2021).

Langerhans cells, which are part of the skin's immune system, reside in the epidermis. These cells contribute to both the immune response against infections and the onset of skin allergies (Karim and Aryani 2021).

The dermis, which is a thick, elastic, and fibrous tissue layer primarily composed of collagen and a small amount of elastin, lies beneath the epidermis. The dermis is the source of skin elasticity and strength. The dermis is home to blood vessels, sweat, and oil glands, nerve endings, and hair follicles (Mohamed and Hargest 2022; Verma et al. 2022).

The hypodermis contains larger nerves, connective tissues, blood vessels, macrophages, and subcutaneous fat. This layer is composed of connective tissues, macrophages, and subcutaneous lipids, and is extensively vascularized by blood vessels (Agarwal and Krishnamurthy 2019).

The hypodermis serves as the main structural support for the skin, acts as a thermal barrier, and controls body temperature to prevent excessive temperatures because it is primarily composed of fat (Abdo et al. 2020).

In addition, it serves as an additional cushioning for the body to safeguard bones and tissues, and is where injections of medications are administered.

**FIGURE 2:**
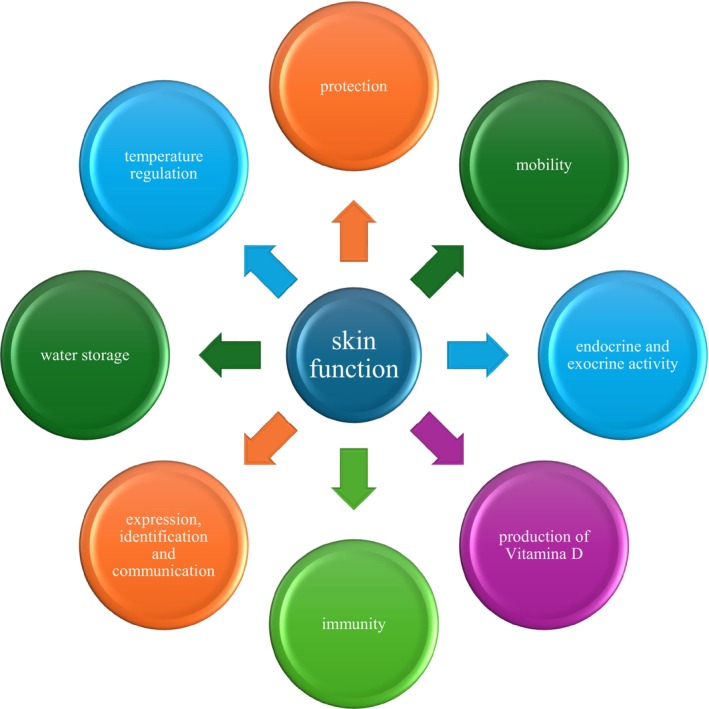
Function of the skin.

The skin performs many tasks (Figure 2). Its ability to act as a barrier between the body's internal organs and the outside world, interact directly with the environment, keep pathogens and dangerous substances out of the body, absorb the shock of mechanical, thermal, or physical stress, shield the body from the damaging effects of ultraviolet radiation (UVR), and withstand water resistance all undoubtedly contribute to its protective qualities. It is an important immunological organ that helps the body fight off infections.

The skin can control body temperature variations in the body. It retains or releases body heat through eccrine sweating, insensible sweating, and dilatory or constrictive alterations in the cutaneous vasculature (Luo et al. 2020).

Because a vast network of nerve cells senses and transmits changes in the surrounding environment, it constitutes a nerve transmission network. In addition to other senses, the receptors are sensitive to temperature, pain, and touch. The motor component of skin innervation includes both involuntary smooth muscles linked to hair follicles and striated muscles directly attached to the skin, such as those involved in facial expression (Kumar 2024). Because the skin is both a target for stress reactions and an instant sensor of them, its capacity to convey emotions is a more community function and an indication of a physical state that plays a significant role in the stress response. As a location for water storage, it also serves another purpose (Mohamed and Hargest 2022). Water is mostly found in the skin's dermis layer and accounts for approximately 18% and 20% of the body's overall volume.

It performs an endocrine function by playing a significant role in vitamin D synthesis. Exposure to UV radiation (endocrine activity) is the main route through which vitamin D is generated (Aguilar‐Shea 2021).

In order to perform its exocrine function, it releases water, urea, and ammonia. Apart from secreting substances such as pheromones, sebum, and sweat, the skin also performs essential immunological functions by secreting bioactive substances like cytokines (Chambers and Vukmanovic‐Stejic 2020).

The skin facilitates smooth movement, which is why the body can move.

## 3 Aging Process

Both inherited and environmental factors contribute to aging. The latter comprises ionizing and UV radiation, physical and psychological stress, and poor nutritional intake.

As we age, our skin's capacity to produce sufficient collagen and elastin to maintain its structure and suppleness decreases. This leads to the development of wrinkles, tone loss, and skin drooping. Skin stem cells are vital for maintaining and repairing skin; however, their potency decreases with age, making skin renewal and self‐healing more difficult (Lee et al. 2021).

Skin aging has two independent processes:

1. Intrinsic skin aging, an inevitable condition and time‐dependent, results from chronological aging and clinically presents as smooth, dry, and thinning skin;
2. Extrinsic skin aging is caused by sun exposure, smoking, pollution, lack of sleep, and poor diet. It presents as wrinkles, pigmented lesions, actinic keratosis, photodamage, and patchy hypopigmentation.

**FIGURE 3:**
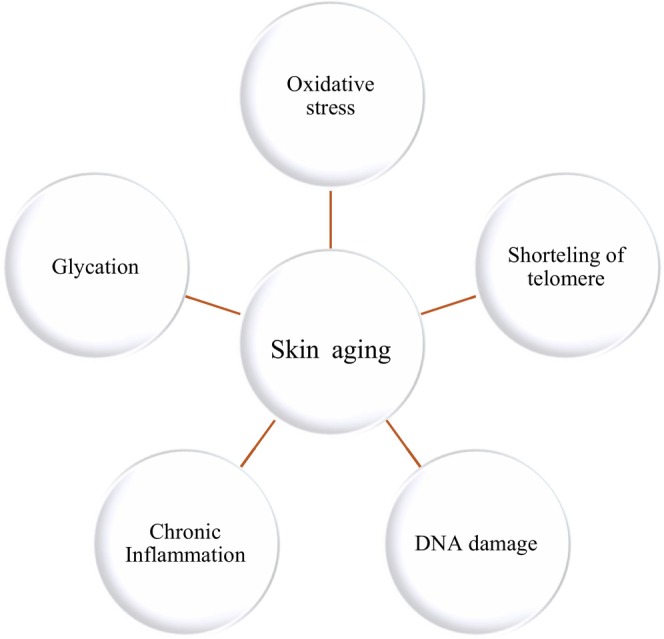
Molecular processes of skin aging.

Recently, researchers have employed various theories to explain the molecular pathways (Figure 3) of skin aging. High‐frequency chromosomal abnormalities, oxidative damage, single gene mutations, persistent inflammation, and cellular aging are some of the contributing causes (Lee et al. 2021; Shin et al. 2019).

Oxidative stress plays a major role in skin damage and skin aging. Its main characteristic is an increase in intracellular ROS (Lim et al. 2022; Papaccio et al. 2022).

Collagen and elastin fibers' structural and functional properties are crucial for preserving skin health; thus, these properties will be a major focus for antioxidant, enzymatic, cellular, molecular, and other variables that regulate this extracellular matrix protein. By suppressing Transforming Growth Factor beta (TGFβ) and limiting procollagen production, activator protein‐1 (AP‐1) is known to be activated by ROS formation. This lowers collagen levels through molecular and cellular cascades (Lephart 2016; Markiewicz and Idowu 2019). Meanwhile, AP‐1 activation:

- Induction of matrix metalloproteinase (MMPs) to activity to break down collagen;
- Triggers nuclear factor kappa‐light‐chain enhancer of activated B cells (NF‐kappaB), a significant inflammatory response activator (Lephart 2016).

Interleukins (ILs), which are released when the inflammatory response starts, increase the amount of ROS produced in the positive feedback loop.

Furthermore, telomere mutation, DNA damage, senescence, and cell death can all result from UV radiation exposure and create ROS (Markiewicz and Idowu 2019). Research is ongoing to determine whether epithelial stem cells with short telomeres have a lower capacity for proliferation (Rossiello et al. 2022).

Although oxidative stress is the primary cause of skin aging, it is not the only mechanism. Other mechanisms include glycation and chronic inflammation (Chen, Zhang, et al. 2022).

The nonenzymatic glycosylation aging theory has been widely accepted by scholars. AGEs are formed during the glycation process when sugars and skin proteins interact. This process alters the structure and function of skin proteins, reducing skin strength and flexibility and hastening the appearance of wrinkles (Chen, Zhang, et al. 2022).

Aging of the skin is associated with low‐grade chronic inflammation. Oxidative stress, DNA damage accumulation, and prolonged exposure to environmental irritants can trigger skin inflammatory pathways. Collagen deterioration and a weakened skin barrier may eventually follow this (Pilkington et al. 2021). These are but a handful of the primary factors behind skin aging; more studies are required to completely understand this complex process, and new discoveries are being made constantly.

## 4 Healthy Diet

A diet high in phytochemicals, proteins, functional peptides, functional oils (rich in bioactive components such as polyunsaturated fatty acids), minerals, probiotics, and vitamins is necessary to enhance the morphological anomalies and functional decrease associated with aging skin (Guo 2023; Ko and Auyeung 2014; Solway 2020). Nutritional imbalance and unhealthy eating habits are related to skin aging.

It is nearly impossible to find a diet or food group that provides all the nutrients your body and skin need, though. It is therefore essential for individuals to have a broad and well‐balanced diet rich in antioxidant‐rich fruits and vegetables, nuts, fatty salmon, and other healthy fats, along with drinking plenty of water. Skin tone can be enhanced, and normal skin cells can form by taking these nutrients (Michalak 2022). Numerous recent studies have demonstrated the anti‐inflammatory benefits of the Mediterranean diet, which is a balanced diet (Tsigalou et al. 2020).

In order to cure or prevent the signs and symptoms of skin aging, functional foods and oral supplements—also referred to as nutraceuticals—are becoming increasingly popular alternatives to conventional dermatological procedures like botulinum toxin, fillers, and aesthetic surgery (Iwatani and Yamamoto 2019).

Antioxidants are widely known for their ability to shield the skin from oxidative damage. Vitamin C protects against photoaging and promotes collagen synthesis. Similarly, higher dietary vitamin E intake has been associated with less wrinkle formation and increased skin suppleness. Fruits, vegetables, and dried fruit are the main ingredients. These exogenous antioxidants have become an excellent means of enhancing the skin's built‐in photoprotective defenses against environmental stresses and preserving skin regeneration (Mumtaz et al. 2021; Rattanawiwatpong et al. 2020; Arangia et al. 2023). Nutrients such as proline, lysine, and vitamin C, which are involved in the manufacture of collagen, are primarily responsible for maintaining skin firmness and durability. Supplemental collagen peptides have been shown to have an effect on skin hydration and suppleness, particularly in elderly individuals (Nobile et al. 2023; Lupu 2020). Nutrient‐rich foods that boost collagen include citrus fruits, almonds, and seafood.

The anti‐inflammatory and antioxidant properties of plant‐derived compounds, such as phytonutrients, flavonoids, polyphenols, and carotenoids, are linked to skin health. Eating meals rich in phytonutrients on a regular basis has been associated with improved skin appearance and decreased risk of photoaging. For example, phenolic compounds, which have anti‐inflammatory and antioxidant qualities, are abundant in apples, oranges, and yellow fruits and vegetables, garlic, onions, chives, and leeks, among many other foods (Monjotin et al. 2022; Interdonato et al. 2023).

Together with necessary fatty acids, omega‐3 polyunsaturated fats also contain anti‐inflammatory properties that may slow down the aging process of the skin. Consuming foods rich in omega‐3 fatty acids has been linked to improved skin barrier function and decreased transepidermal water loss, both of which indicate increased hydration and undamaged skin (Sorokin et al. 2023). Examples of these foods include almonds, flax, and chia seeds, as well as salmon, mackerel, herring, trout, and clams.

As cofactors in several enzyme systems and fundamental components of the skin's structure, minerals and vitamins are vital micronutrients that humans cannot produce on their own and must be received through diet. Macroelements such as calcium, phosphorus, magnesium, potassium, sodium, and iodine, as well as microelements like sulfur, manganese, zinc, iron, iodine, chlorine, copper, and selenium, are necessary for a balanced diet (Cao et al. 2020; Shamloul et al. 2019; Kokande 2024).

Probiotics may also aid in moisturizing, removing body odor, anti‐aging, reducing wrinkles, and skin whitening. There are numerous methods in which prebiotics and probiotics can enhance, maintain, and heal the skin microbiome. For instance, bacterial fermentation can intensify the anti‐photoaging effects of agastache rugosa leaves, and specific probiotic preparations may aid in treating aging skin (Seo et al. 2019). Yogurt, fermented foods (kefir, tempeh, sauerkraut), and any high‐fiber food—vegetables and fruit—all contain probiotics. Whole grains like brown and spelt rice, legumes like chickpeas and lentils, beans, vegetables like asparagus, artichokes, chicory, onion, garlic, bananas, yogurt, and fermented milk (kefir, for instance) are all good sources of prebiotics (Chen, Zhao, et al. 2022).

A small or insufficient amount of estrogen (known as phytoestrogens) reduces the thickness, elasticity, and collagen of the skin and makes it resistant to oxidative stress. Additionally, it causes wrinkles and dryness to worsen (Lephart 2021). The appearance, composition, and general health of the skin are known to vary significantly in cases of estrogen insufficiency, especially during or after menopause (Lephart and Naftolin 2021). Numerous fruits and vegetables, such as onions, peanuts, chickpeas, peas, whole grains, beans, lettuce, lentils, and others, readily contain these. Recent research has examined the potential of some nutrients to enhance skin health and slow down the aging process. For instance, pistachios, cashews, and ACAI berries have anti‐inflammatory, antioxidant, antibacterial, antiproliferative, and astringent qualities due to the minerals and chemicals they contain that have various medicinal effects. Several in vivo and in vitro studies have demonstrated that these actions are primarily focused on combating inflammation and oxidative damage (Di 2022). These are only a handful; a comprehensive list can be found in Table 1.

**TABLE 1:** Nutrients that slow down skin aging.

Nutrient	Activity	References
*Moringa oleifera*	Packed with antioxidants, it prevents free radical damage	Sławińska and Olas (2023)
*Cranberry*	The polyphenols it contains inhibit the formation of final glycation products in collagen	Chang et al. (2022)
Orange Juice from *Citrus sinensis*	It has a preventive effect against ROS generation and inflammation caused by the presence of flavonoids	Fusco et al. (2017)
*Açai berry*	Being abundant in antioxidants and vitamins A and C, they are excellent anti‐aging ingredients that promote collagen formation, keeping skin smooth and devoid of wrinkles	Siracusa et al. (2022)
*Pistachio, Cashew nuts*	UVR‐induced skin damage is a change that polyphenolic antioxidants prevent due to ROS overproduction	Di (2022)
*Aloe Vera*	Enhances suppleness and wrinkles, and raises type I procollagen gene expression.	Xie et al. (2022)
*Spirulina*	Improves collagen and antioxidant activity while reducing inflammation and collagen degradation, thereby postponing the appearance of skin aging	Sharafeldein et al. (2023)
*Rosmarinus officinalis* extracts	Possesses strong antioxidant properties and acts to reduce inflammatory cytokine and MMP‐1 transcriptional and basal levels	Sharafeldein et al. (2023)
*Extra virgin olive oil*	Reduces oxidative damage in keratinocytes by enhancing mitochondrial function	Yazihan et al. (2020)
*Green tea*	Lowers ROS levels, safeguards collagen formation, lowers MMP secretion, and controls the NF‐κB, AP‐1, and MAPK signaling pathways	Wang et al. (2019)
Fatty fish *(salmon and tuna)*	Omega‐3 fatty acids may aid in reducing inflammation and are associated with a robust skin barrier. elevated selenium levels (a mineral antioxidant)	Muzumdar and Ferenczi (2021)
*Chocolate and Cocoa*	Includes antioxidant‐acting flavanols. According to several studies, it might enhance skin	Tan et al. (2021)
Vegetables with the highest carotenoid content *(carrots, pumpkin, sweet potatoes)*	A diet high in carotenoids (such as carotene and lycopene) may protect the skin against UVR	Balić and Mokos (2019)
Vegetables with the highest vitamin C content *(leafy greens, bell peppers, tomatoes, broccoli)*	As a potent antioxidant, vitamin C also plays a crucial role in collagen production	Boo (2022)
*Tomatoes*	High levels of lycopene and vitamin C	Ambarwati et al. (2022)
*Flax seed*	Include lignans, which are antioxidants that aid in the fight against free radicals. Moreover, they contain a high level of alpha‐linolenic acid (ALA), an omega‐3 that promotes a healthy skin membrane	Patel (2021)
*Pomegranates*	Abundant in antioxidants, including lignans, tannins, phenolic acids, and flavonoids, which aid in skin regeneration and protect the skin from UVR damage	Chan et al. (2021)
*Avocados*	Their strong antioxidant concentration may help combat free radicals that damage and age the skin, whereas their high monounsaturated fat content may support a healthy skin membrane and promote healthy skin	Flores‐Balderas et al. (2023)
Chickens and eggs	Hydrolyzed collagen peptide content enhances skin firmness, elasticity, and moisture	Wang et al. (2023)
*Tofu*	Because isoflavones replace estrogen, they improve overall skin quality, particularly in postmenopausal women	do Prado et al. (2022)

## 5 Unhealthy Diet

**FIGURE 4:**
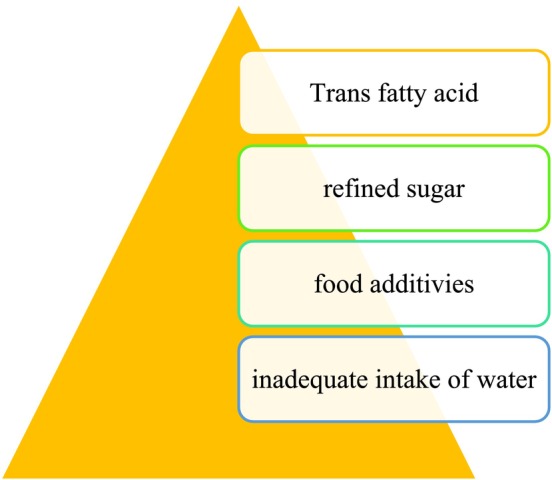
Unhealthy diet.

We examined the ways that a balanced diet can prevent aging and promote skin health, as well as the new nutrients that can support this. However, there is also an opposing viewpoint (Figure 4).

Our time frequently encourages us to consume foods high in trans‐fatty acids, refined sugar, food additives, and insufficient amounts of water; these are a few instances of what is regarded as an unhealthy diet (Laura 2019).

In terms of hydration, dehydrated skin usually exhibits an uneven tone and complexion, roughness, dryness, loss of elasticity, tension, and itching. This phenomenon occurs because dehydration lowers the stratum corneum's water content, which interferes with enzyme functioning (Park et al. 2021).

The European Food Safety Authority defines adequate water intake as 2.0 L/day for women and 2.5 L/day for men (Nakamura et al. 2020).

There is evidence in the literature that dietary fat intake and skin aging are related, with high dietary fat intake having a negative impact on the appearance of skin aging (Muzumdar and Ferenczi 2021). Diets high in polyunsaturated fats have been associated with a modestly elevated risk of skin cancer (Bojková et al. 2020), as well as a slower rate of skin healing and higher levels of oxidative stress and inflammatory reactions. Most dietary fatty acids with trans double bonds come from industrial sources, particularly when edible oils are partially hydrogenated with unsaturated fatty acids to create saturated fats. Trans fats can be found in significant amounts in various foods, including cakes, cookies, animal products, margarine, chips, and French fries. On the other hand, decreased wrinkles, age‐related dryness, and skin atrophy are linked to a low‐fat diet and high linoleic acid levels (Wang and Wu 2019).

Consumption of carbohydrates is another factor to consider. A balanced diet requires a certain amount of carbohydrates. Increased signs of skin aging are associated with refined sugar consumption (Cao et al. 2020). The impact of refined carbohydrates on skin aging is mostly caused by the covalent cross‐linking of the two collagen strands (Berticat et al. 2020). There is a small but significant pro‐acnegenic effect associated with the use of refined carbohydrates, high‐glycemic index foods, and high‐glycemic load foods (Berticat et al. 2020).

Recent studies have suggested that cutting calories or fasting may have positive effects on skin (Mehdi et al. 2021).

Caloric restriction slows the glycation rate of skin proteins in rats, preventing the production of AGEs linked to aging in skin collagen (Cefalu et al. 1995). Age‐dependent glycoxidation product buildup is reduced by calorie restriction (Okouchi et al. 2019).

Drinking too much alcohol is also seen negatively because it damages the skin barrier function, changes the permeability of the skin, and encourages the growth of keratinocytes (Cao et al. 2020).

## 6 Bioactive Ingredients Useful for Preventing Skin

In response to health requirements, food manufacturers have recently begun to produce “functional foods,” which are defined as food items with added positive health benefits (Granato et al. 2020).

They seem to be the same as traditional meals aside from their basic nutritional benefits, but in contrast to traditional diets, they have been shown to have physiological benefits and can reduce the risk of chronic diseases (Cencic and Chingwaru 2010).

Probiotics found in food and its products, for instance, have been shown to reduce the signs of skin aging (Permatasari 2021).

Nutraceuticals—whose application is predicated on the idea of “food as medicine”—should not be confused with functional foods. In fact, functional foods are food‐based items that are advertised as having therapeutic or health‐promoting qualities. They are frequently used to treat particular health problems or as a dietary supplement (Cencic and Chingwaru 2010; Pang 2023; Xu et al. 2023).

The newest trend in the beauty market is nutraceuticals, which emphasize skin care, hair, and nails. Because people are increasingly aware of what they put into their bodies and seek natural goods that can improve their appearance and health, this trend agrees with contemporary society. (Laura 2019). Nutricosmetics are products and ingredients that act as dietary supplements intended to preserve skin beauty. These consist of edibles and beverages with functional properties, such as snack bars, baked products, and other treats augmented with chemicals that offer therapeutic or esthetic benefits. The skin's natural hydration is enhanced by various drinks and foods, such as enriched water, coffee, herbal teas, and fruit and vegetable juices (Laura 2019). Below is a summary of the active ingredients useful for preventing skin aging that are used in nutricosmetics (Table 2).

**TABLE 2:** Bioactive compounds useful in nutricosmetics.

Bioactive compound	Role in skin aging	References
Peptides: Collagen peptides	Oral collagen peptide supplements greatly improve skin hydration and cause the breakdown of the dermal collagen network, which promotes the synthesis of collagen and glycosaminoglycans	Pu et al. (2023)
*Selenium*	Oxidative stress is a cofactor of glutathione peroxidases and thioredoxin reductases and aids in the removal of hazardous lipid hydroperoxides, hydrogen peroxide, and peroxynitrite. Thus, selenium protects against oxidative stress and damage caused by these processes	Barchielli et al. (2022)
*Vitamin E*	Capacity to smother singlet oxygen of reactive oxygen species (ROS), inhibit lipid peroxide‐free radical synthesis in an inactive state, and prevent chain reactions that harm cells.	Chaudhary et al. (2023)
*Hyaluronic acid*	It is an important component of dermis metabolism and a component of the skin's extracellular matrix; supplementation improves skin hydration and elasticity while lowering the depth of wrinkles and roughness	Keen (2017)
*Zinc*	Contributes significantly to the morphogenesis, maintenance, and repair of the skin. It is a necessary cofactor for many metalloenzymes (MMPs), RNA polymerases, superoxide dismutase, and metallothionein	Haftek et al. (2022)

## 7 Conclusions

Numerous interventional studies are necessary to fully understand the complex and contradictory relationship between dietary problems and skin aging. Antioxidant systems within cells provide the best defense against the aging of the skin. In conclusion, there are different nutrients that have different yet related impacts on skin structure and function, contributing to the complex relationship between nutrition and skin aging. A diet high in antioxidants, omega‐3 fatty acids, nutrients that build collagen, foods that promote moisture, and phytonutrients can help people maximize skin health and reduce the appearance of obvious indications of aging. To clarify ideal food habits and their long‐term impact on skin aging, considering individual differences and lifestyle factors, more research is necessary. Many scientists working in the domains of nutrition and dermatology find it fascinating how diet affects the aging process of the skin. This subject is crucial because modifying one's diet is one of the most effective and realistic ways to stop or slow down the aging and damaging processes of the skin. Consuming antioxidants on a daily basis, primarily from fruits and vegetables, is thought to help scavenge reactive oxygen species and prevent aging and photodamage of the skin. It remains unclear whether nutrition affects how skin functions or matures. Several intervention studies are required to provide an answer.

## Author Contributions

**Nicla Tranchida:** writing – review and editing (equal). **Francesco Molinari:** writing – original draft (equal). **Gianluca Antonio Franco:** writing – original draft (equal). **Marika Cordaro:** conceptualization (equal). **Rosanna Di Paola:** project administration (equal).

## Conflicts of Interest

The authors declare no conflicts of interest.

## Data Availability

The authors have nothing to report.
